# Code-Sharing in Cost-of-Illness Calculations: An Application to Antibiotic-Resistant Bloodstream Infections

**DOI:** 10.3389/fpubh.2020.562427

**Published:** 2020-11-27

**Authors:** Nichola R. Naylor, Kazuto Yamashita, Michiyo Iwami, Susumu Kunisawa, Seiko Mizuno, Enrique Castro-Sánchez, Yuichi Imanaka, Raheelah Ahmad, Alison Holmes

**Affiliations:** ^1^NIHR Health Protection Research Unit in Healthcare Associated Infections and Antimicrobial Resistance, Imperial College London, London, United Kingdom; ^2^Department of Healthcare Economics and Quality Management, Kyoto University, Kyoto, Japan; ^3^Department of Infectious Disease, Imperial College London, London, United Kingdom; ^4^School of Health Sciences, Division of Nursing, City, University of London, London, United Kingdom; ^5^School of Health Sciences, City, University of London, London, United Kingdom; ^6^Imperial College Healthcare NHS Trust, Hammersmith Hospital, London, United Kingdom

**Keywords:** cost, length of stay, antibiotic resistance, code-sharing, *Staphylococcus aureus*

## Abstract

**Background:** More data-driven evidence is needed on the cost of antibiotic resistance. Both Japan and England have large surveillance and administrative datasets. Code sharing of costing models enables reduced duplication of effort in research.

**Objective:** To estimate the burden of antibiotic-resistant *Staphylococcus aureus* bloodstream infections (BSIs) in Japan, utilizing code that was written to estimate the hospital burden of antibiotic-resistant *Escherichia coli* BSIs in England. Additionally, the process in which the code-sharing and application was performed is detailed, to aid future such use of code-sharing in health economics.

**Methods:** National administrative data sources were linked with voluntary surveillance data within the Japan case study. R software code, which created multistate models to estimate the excess length of stay associated with different exposures of interest, was adapted from previous use and run on this dataset. Unit costs were applied to estimate healthcare system burden in 2017 international dollars (I$).

**Results:** Clear supporting documentation alongside open-access code, licensing, and formal communication channels, helped the re-application of costing code from the English setting within the Japanese setting. From the Japanese healthcare system perspective, it was estimated that there was an excess cost of I$6,392 per *S. aureus* BSI, whilst oxacillin resistance was associated with an additional I$8,155.

**Conclusions:** *S. aureus* resistance profiles other than methicillin may substantially impact hospital costs. The sharing of costing models within the field of antibiotic resistance is a feasible way to increase burden evidence efficiently, allowing for decision makers (with appropriate data available) to gain rapid cost-of-illness estimates.

## Introduction

Health economics and outcomes research related to infectious disease often needs to take into account unique factors that can lead to increasingly complex statistical and economic methodology (1). Cost-effectiveness models of interventions related to infectious disease should (generally) account for infectious disease transmission dynamics (2), whilst costing studies related to healthcare-associated infections should account for factors such as time dependency bias. Time dependency bias describes a bias that arises when the time of infection isn't fully taken into account when attributing hospital costs to a condition (1). For example, if a full hospital stay was attributed to a methicillin-resistant *Staphylococcus aureus* (MRSA) infection, but the patient only contracted MRSA after being in hospital for 10 days, you are wrongly attributing 10 days of hospital costs to that MRSA infection. Estimating the cost of such infections accurately is key to ensuring robust cost parameters are utilized in cost-effectiveness analyses. Current literature has focused on estimating the impact of methicillin resistance, as opposed to also estimating the burden of other antibiotic resistance profiles relative to *S. aureus* infections (3, 4).

Accounting for time dependency bias, whilst also adjusting for other key factors like patient age and/or comorbidity, requires the use of models such as sub-distribution hazard models (5) or adjusted multistate models (6). In order to apply such techniques, a flexible data analysis software environment is needed (6). Similarly, building cost-effectiveness models which account for transmission of infections, potentially between patient populations and environments, requires more flexibility in the model building process. These factors have played a part in the increase in the use of R, an open-source software environment that allows users to build complex, health economics models directly, or easily adapt the previous work of other users (7–9).

There are two main ways in which health economists can utilize and build upon the work of previous colleagues when constructing models in R; the first is through downloading “packages” directly from the host of R code (“The Comprehensive R Archive Network”) (10), the second is downloading available code from code-sharing sites (11). Packages are, in essence, downloadable scripts of code performing defined functions (such as de-duplicating datasets), with documentation available online explaining the usability of such functions (10). Code shared via open-access, code-sharing websites can provide the same service, however can be more informal. Additionally, researchers may choose to share code on such sites not as a way to share defined “reproducible” functions for future analyses, but rather to be transparent in the data analysis or modeling procedures used in relevant published manuscripts.

The practice of using code-sharing sites for collaboration and transparency should, theoretically, reduce time spent on duplication of basic code, increase efficiency in building health economic models within the field of infectious disease and increase robustness of such models (due to potential critique through increased transparency). As the use of R for health economic models continues to grow, and with the addition of health economics package collations available online (12, 13), we wanted to show the potential advantage of code-sharing in health economics applied to infectious disease.

Both Japan and England have large, infection surveillance and hospital administrative data sets (9, 14). Therefore, this report discusses the process and subsequent results of an international collaboration in which Japan-based health economists estimated the hospital cost of bloodstream infections using England-based equivalents' R code (9), shared through GitHub (15). The objectives of this brief report were to; (i) describe the code sharing process used to estimate cost of infections across two different, high-income country settings; and (ii) describe the top-line results of the analysis in Japan for a range of antibiotic resistance profiles.

## Methods

### Process Methodology

Research was previously conducted to estimate the health and cost burden of antibiotic-resistant and antibiotic-susceptible *Escherichia coli* bloodstream infections in the English secondary care setting, using national administrative and surveillance datasets (9), this will be referred to as “the English study.” As some of these infections occur during a patient's hospital stay, time dependency bias had to be taken into account, and as such multistate models were built to estimate excess length of hospital stay (LoS) associated with *E. coli* bloodstream infections (1).

Multistate models estimate LoS as a function of (i) the number of people within each health state (such as number of patients in the infected state) and (ii) the number of transitions between health states (such as number of patients moving from infected to discharged) for specified time intervals (such as days) (16). The English study utilized R software to construct these models, specifically using the “etm” and the “mvna” packages (17, 18). As these R packages allowed for a maximum of one exposed group and non-exposed group to be compared at a time, models were constructed that first compared infected with non-infected patients, and then compared antibiotic resistant/intermediate infections with antibiotic susceptible infections for different antibiotics of interest (9).

The related code was subsequently deposited (open-access) to GitHub (15). The relevant GitHub repository included the R scripts used within the data analysis on the English datasets, a data dictionary detailing the key variable definitions and a codebook which describes what the R scripts intend to do. This code was then downloaded by colleagues within the Japan study, adapted where needed, and utilized on Japanese hospital data to estimate the cost burden of antibiotic-resistant *S. aureus* bloodstream infections. This will be referred to as “the Japanese study.”

To begin the code application within the Japanese study, the code was downloaded and tested by health economists. Subsequently, a meeting was held with colleagues across the partnering institutions to go through the code and its application within the Japanese study. Whilst a face-to-face (or virtual) meeting is not a necessity, the authors found it an efficient way of dealing with nuances of the code application to different data sources.

### The Japanese Study

Copies of datasets from participating hospitals of The Japan Nosocomial Infections Surveillance (JANIS) system, a Japanese governmental surveillance system, were utilized as the data source for this analysis. JANIS is a voluntary surveillance system which covers around 30% of Japanese hospitals (19). These bacterial surveillance data collected from hospitals (JANIS dataset) were linked to administrative data; Diagnosis Procedure Combination (DPC) data, also collected from these hospitals to obtain admission, discharge, and patient data [JANIS-DPC database (4)]. The Ethics Committee, Graduate School of Medicine, Kyoto University approved the study (reference - R0577).

Acute care hospitals (most of which are educational hospitals) were included in this analysis, including private, public, and university hospitals throughout Japan. This is a similar setting to the English study, which was based in acute care National Health Service (Foundation) Trusts. Adult, patient data on *S. aureus* bloodstream infections were extracted from the JANIS dataset between April 2014 and March 2016. This represented two Japanese, fiscal years. Definitions of antibiotic resistance were in line with the JANIS data definition, which is line with governmental guidance (20). Antibiotic resistance impact was investigated in relation to first-generation cephalosporins, carbapenems, gentamicin, fluroquinolones, and penicillin (including methicillin and oxacillin), as these are important classes with resistance case numbers >1,000. Additionally, oxacillin was selected by the Japanese study as a key antibiotic to test individually. “Not-tested” was included in our non-exposed controls, allowing for use of all available data and consistency with the English study (9).

**Figure 1:**
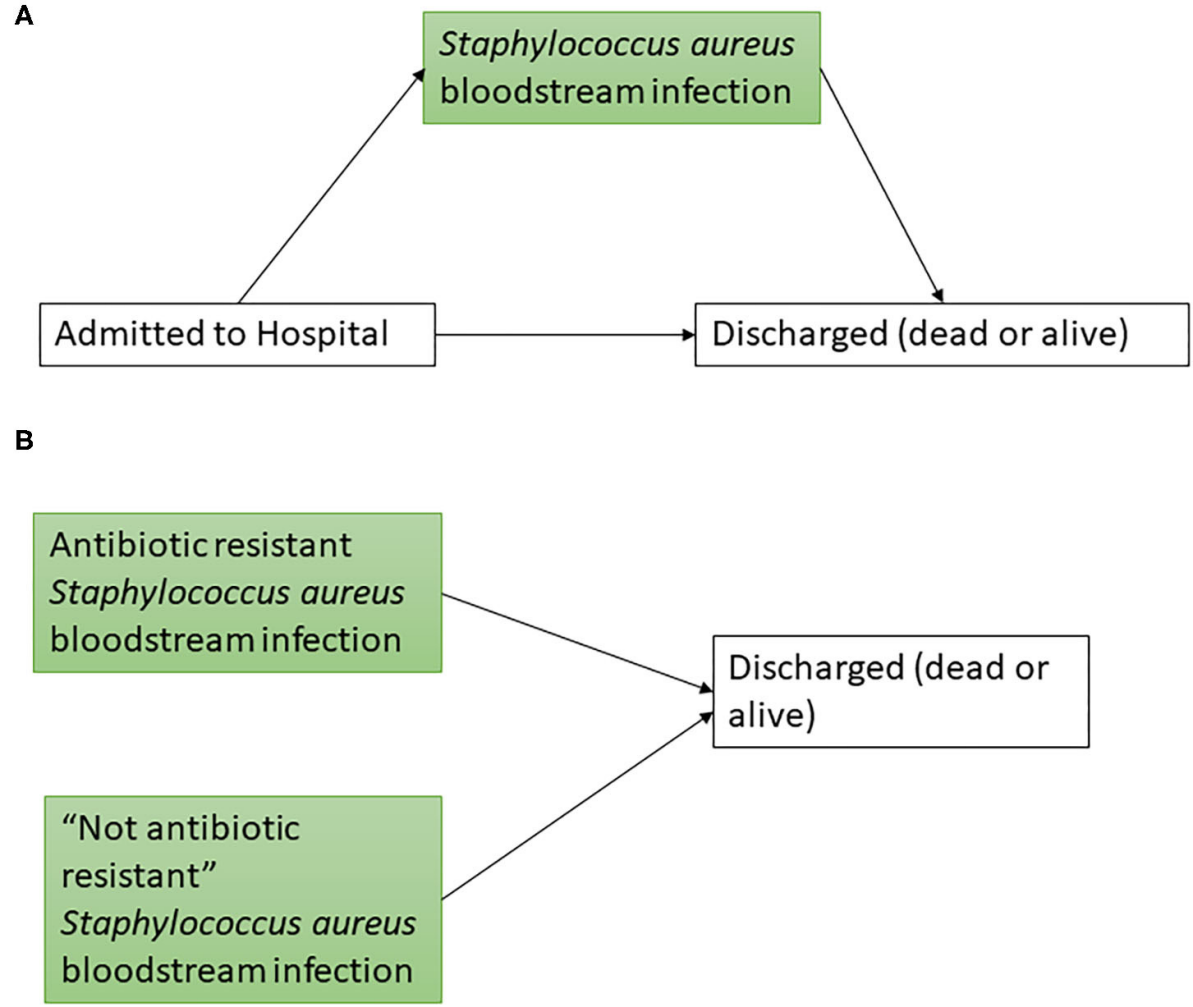
Multistate models used to estimate excess length of stay. Boxes represent possible health states (green boxes representing *Staphylococcus aureus* states) and arrows represent potential transitions between states. For **(A)** the exposure group is *Staphylococcus aureus* bloodstream infections. For **(B)** a separate model was constructed for each antibiotic exposure group of interest, whereby healthcare-associated infections entered the model at time of infection. “Not antibiotic resistant” included susceptible and not tested (as defined by the data source used).

Multistate models were used to estimate the excess LoS of *S. aureus* bloodstream infections, as done in previous analyses (3, 8, 9). Figure 1 depicts the structure of these. Cumulative transition hazards representing the movement between states were calculated using the data provided. These were then used to estimate “expected LoS” on each day (t) (16, 21). These estimates for each day (e.g., expected LoS for infected patients minus expected LoS for non-infected patients on *t* = 2) are then averaged across all days of the study period (weighted by frequency of events) to get an estimate of average excess LoS across the groups tested (7). Artificial censoring was used to reduce the impact of outliers on multistate model results (3). The artificial censoring time was moved from 45 days (from the English study) to 90 days (for the Japanese study), due to the longer average inpatient LoS for Japanese hospital patients. For example, a recent OECD report has stated that average LoS for acute care in Japan in 2017 (16.2 days) was much higher than in the UK (6.9 days, respectively) (22).

To estimate costs from the healthcare system perspective, the unit cost of an excess bed day was applied to excess LoS estimates. The most applicable cost found was the World Health Organization (WHO) estimation of a cost per inpatient bed day for Japan [514.26 in 2010 international dollars (I$)], which was estimated as part of the WHO-CHOICE estimates of “cost for inpatient and outpatient health service delivery” (23). This 2010 cost was converted to Japanese Yen using purchasing power parity (PPP) (24), inflated to 2017 costs using the Gross Domestic Product implicit price deflator (25), then converted back to I$ using PPP (24). This gave an estimate of $551 per day (2017 I$). This process of cost conversion is in line with guidance that has been previously published (26, 27).

## Results

### Process Results

Researchers from the English study cleaned the R script code, wrote a corresponding data dictionary and a relevant codebook to explain the R scripts. These were then uploaded to a code sharing website (15). Researchers in the Japanese study downloaded this code and began to adapt according to their exposures of interest and the data available. For example, variable names within the cleaning code of the English study needed to be adapted to match the variables names in the linked JANIS-DPC database. Colleagues leading the Japanese study were familiar with the datasets, enabling an understanding of how to adapt data formats to fit into the coded structure fairly quickly. Harmonizing data structures is key in applying code that was built for other analyses, and determination as to whether key variables are present in the application dataset is needed as a first step.

R scripts that created time-dependent datasets (i.e., accounted for the time between admission and infection for hospital-onset infections), created multistate models to estimate excess LoS were downloaded, adapted, and run in the Japanese study. Subsequently, licensing information for the code was added to the English study code (15), and should be added in future coding uploads of this nature, to reduce legal ambiguity on the source code (28).

Clear annotation of code, and consistent labeling of particular processes was noted as highly useful for the Japanese study when utilizing the English study code. Throughout the data cleaning and analyses, the data dictionary and codebook were noted as being instrumental in aiding the adaptation of variable names and R code processes to fit the Japanese data.

This process led to time-efficient cost estimates for *S. aureus* being available, with reduced time spent on initial data cleaning and basic analysis coding for the Japanese study.

### The Japanese Study Results

4,017 *S. aureus* bloodstream infection inpatient spells were included in the analysis. To estimate the excess LoS of these infections, these spells were compared to 1,215,119 patient spells which were not related to *S. aureus* bloodstream infections. Descriptive statistics are presented in Table 1, in which you can see over 25% of exposed patient spells resulted in in-hospital mortality, compared to <5% in the non-exposed group.

**Table 1:** Descriptive statistics of exposed and non-exposed patient hospital spells.

**Descriptor**	**Characteristic (measure)**	**Non-“*Staphylococcus aureus* BSI”**	***Staphylococcus aureus* BSI**
Sample size	Number of hospital spells	1,215,119	4,017
Gender	Female	575,615 (47.4%)	1,586 (39.5%)
Median age	Median age in years (IQR)	70 (58–79)	77 (66–85)
Modified Elixhauser Comorbidity Index (29)	Mean (SD)	5.17 (6.63)	7.64 (7.06)
Average length of stay	Median days in hospital (IQR)	8 (4–17)	34 (16–63)
Mortality	In-Hospital mortality (%)	4.8%	27.6%

*BSI, bloodstream infection; IQR, interquartile range; SD, standard deviation. Descriptive statistics were summarized, with median and interquartile ranges (or mean and standard deviation) used for continuous variables, and proportions (represented by percentages) used for categorical variables*.

**Figure 2:**
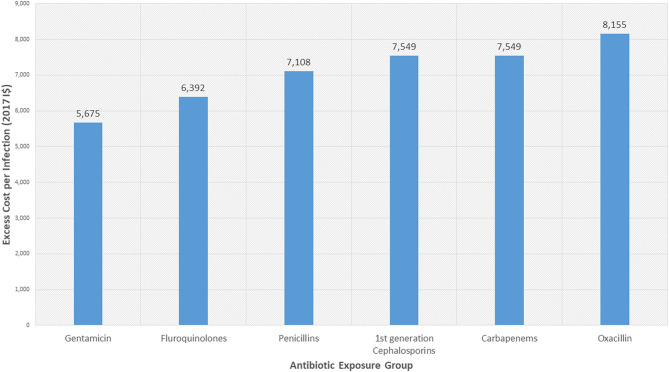
Excess hospital costs associated with antibiotic resistance in *Staphylococcus aureus* bloodstream infections. Costs presented are in 2017 International Dollars (I$). Costs presented are those associated with excess length of stay. The exposure groups are patients with *S. aureus* bloodstream infections that are resistant to the stated antibiotic groups. These are compared to patients with *S. aureus* bloodstream infections that are not resistant to the respective antibiotic groups. Note the penicillin category includes methicillin and oxacillin. Oxacillin was selected by the Japanese study as a key antibiotic to test individually in addition to this. Ordering is based on ascending cost estimates.

Initial results showed that *S. aureus* bloodstream infections, when accounting for time dependency alone, were associated with an excess length of hospital stay by an average of 11.6 days. This increase in hospital stay translated into an excess cost of I$6,392 per infection, when applying the unit cost of a bed day (as described in the methods section). Table 2 highlights the excess LoS estimates found. Resistance to all tested antibiotics was associated with an excess LoS of over 10 days. The resistance exposures that had the largest absolute association were oxacillin (14.8 excess days), first generation cephalosporin (13.7 days) and carbapenem (13.7 days) resistant exposures (comparative to their relevant non-exposed cases, respectively). The translation of this into estimates of monetary impact can be seen in Figure 2. The range of the excess cost per infection associated with resistance was estimated to be from I$5,675 (for gentamicin resistance) to I$8,155 (for oxacillin resistance).

**Table 2:** Excess length of stay estimates for *Staphylococcus aureus* bloodstream infections according to resistance profiles.

**Exposure group (number of hospital spells)**	**Non-exposure group (number of hospital spells)**	**Excess length of stay (in Days)**
Staphylococcus aureus (SA) BSI (*n* = 4,017)	Non-infected controls (*n* = 1,215,119)	11.6
SA BSI resistant to 1st generation Cephalosporins (*n* = 1,393)	SA BSI not resistant to 1st generation Cephalosporins (*n* = 2,624; 2,283 susceptible and 341 not tested)	13.7
SA BSI resistant to Carbapenems (*n* = 1,362)	SA BSI not resistant to Carbapenems (*n* = 2,655, 2,340 susceptible and 315 not tested)	13.7
SA BSI resistant to Gentamicin (*n* = 1,334)	SA BSI not resistant to Gentamicin (*n* = 2,683, 2,505 susceptible and 178 not tested)	10.3
SA BSI resistant to Fluroquinolones (*n* = 1,627)	SA BSI not resistant to Fluroquinolones (*n* = 2,390, 2,273 susceptible and 117 not tested)	11.6
SA BSI resistant to Penicillins (*n* =2,751)	SA BSI not resistant to Penicillin (*n* = 1,266, 1,250 susceptible and 16 not tested)	12.9
SA BSI resistant to Oxacillin (*n* = 1,186)	SA BSI not resistant to Oxacillin (*n* = 2,831, 1,633 susceptible & 1,198 not tested)	14.8

*All estimates adjust for time dependency only. BSI, bloodstream infection; n, number of spells relating to that exposure/non-exposure; SA, Staphylococcus aureus*.

## Discussion

This work highlights the potential for code sharing to reduce research burden in health economics across international settings. This report details key things to consider in the sharing of costing models, namely; discussing the appropriateness of the data structures and variables initially, ensuring appropriate licensing is in place and being upheld, providing and using in-depth supporting documents, and (even if a formal collaboration is not feasible) communication between the code's original authors and those applying code is necessary to reduce mistakes in code interpretation and/or application. Code that was written to analyze English hospital data (9, 15), was used directly on Japanese hospital data, producing time-efficient cost estimates relating to *S. aureus* bloodstream infections. This suggests such code [which is currently available open-access (15)] may be useful in other healthcare systems in estimating the impact of antibiotic resistance.

It was estimated that *S. aureus* bloodstream infections was associated with patients staying in hospital for an extra 12 days on average. Antibiotic resistance was estimated to be associated with an excess LoS of between 10 and 15 days, for tested exposure groups. Previous research within the Japanese setting that estimated the burden of MRSA, based on antibiotics prescribed, estimated an excess LoS of 51 (95% Confidence Interval; 30–88) days for those on “anti-MRSA”-antibiotics, 16 (9–30) days for those on “non-anti-MRSA” antibiotics and 6 (3–12) days for non-infected patients (4). However, the case definitions were different so it is difficult to make direct comparisons to our results.

Estimates of excess length of stay from resistance-related exposures of interest ranged from around 10–15 days in the Japan case. This is much higher than those estimated in the English study (ranging from roughly 0.5–1.5 days). However, this is in line with general differences seen in hospital LoS across the two studies, with the non-infected controls for Japan and England having a median LoS of 8 and 0.5 days, respectively (4, 9). Additionally, as highlighted in the methods, this is in line with previous international comparisons of inpatient LoS (22). Cited potential reasons for such international variation include differences in bed supplies and differences in hospital payment systems (22).

### Strengths and Limitations

Getting rapid, initial results in estimating the burden of antimicrobial-resistant infections, as in the case presented here, can be important for deciding treatment policy on a regional or local level. The excess LoS and cost results presented in this report provide initial estimates of the absolute effects associated with a variety of antibiotic exposures. These estimates highlight the need for future primary and secondary research in *S. aureus* bloodstream infections to investigate the impact of different antibiotic susceptibility profiles (such as oxacillin), not just methicillin, on patient outcomes.

The costs are derived using an excess bed day cost (so do not account for further drug or other associated medical costs). Though this is in line with previous literature (3, 9). The cost of a bed-day was taken from WHO-CHOICE, which is a modeled cost estimated that was based on a global analysis (23). However, the authors were not aware of other, usable estimates within the literature for this setting. Another bias that may result in conservative estimates is that bloodstream infections resistant to exposures of interest (but not tested) could be wrongly placed within the non-exposed category. However, this was preferred to dropping non-tested cases or grouping them with the exposed category, the latter of which could lead to overly cautious estimates in terms of resistance-associated excess LoS. Additionally, these estimates are based on acute care hospital data, and therefore may not be applicable to other healthcare settings.

Uncertainty has not been estimated through the application of techniques such as bootstrap sampling, however, such processes do require more time and computing resources. Statistical significance is therefore not determined for the outcomes presented in this brief research report. Additionally, the underlying excess LoS results reported are estimates adjusting for time dependency alone. Therefore, for more robust excess cost estimates in the future, patient covariates should be taken into account and uncertainty intervals calculated using similar methods as applied in the English study (9). Though limited by the aforementioned factors, the estimates presented here are important regarding Japanese health policy, with the current and potential future burden *S. aureus* bloodstream infections being a major cause for concern in this setting (14).

Processes such as the one described in this report could reduce the “cost of information” when analyzing the value of additional information in health economic evaluations. Though seemingly simple, this process of code sharing and transparency between health economists could lead to more efficient research, more cost evidence, cost-effectiveness evidence and therefore, theoretically, more efficient resource allocation decisions. This is particularly relevant in the field of antimicrobial resistance. There have been calls for more robust epidemiological and health economic estimates utilizing data (30). The sharing of code amongst health economists within this field, and in health economics in general, could help reduce research waste and increase collaboration. The call for open science can be extended to related manuscripts; with an appeal for open-access versions of cost-of-illness manuscripts which describe in more detail the methods and results, for example through author or institutional websites (if not an open-access article in itself).

## Conclusion

Antibiotic resistance (as defined across different antibiotic classes), on average, was associated with an additional healthcare system cost of between I$5,675 and I$8,155 per *S. aureus* bloodstream infection in Japan. These estimates were calculated using reduced resources due to the code-sharing practices described in this report. Such estimates can be used in future budget and research priority setting, whilst such code-sharing practices can reduce future research burden.

## Data Availability Statement

The linked JANIS-DPC dataset analyzed for this study is not fully available due to patient identifiable data being present. However, JANIS does provide surveillance data in its open report available. Requests to access the datasets should be directed to https://janis.mhlw.go.jp/english/about/index.html.

## Ethics Statement

The studies involving human participants were reviewed and approved by The Ethics Committee, Graduate School of Medicine, Kyoto University approved the study (reference - R0577). Written informed consent for participation was not required for this study in accordance with the national legislation and the institutional requirements.

## Author Contributions

KY and SK conducted the analysis and NN wrote the original code adapted by KY. NN wrote the first manuscript draft. NN, KY, and SK wrote up the results and interpreted the initial results. RA, MI, and SM provided results interpretation guidance. YI provided acquisition of data used. SM and MI provided administrative support for this study. YI, EC-S, MI, SK, RA, and AH were involved in obtaining funding for this study and for time used within this study. AH, YI, and RA provided supervision for this project. All authors were involved in the initial conceptual idea for the study through attendance of an initial collaboration meeting and were involved in drafting the manuscript. All authors contributed to the article and approved the submitted version.

## Conflict of Interest

The authors declare that the research was conducted in the absence of any commercial or financial relationships that could be construed as a potential conflict of interest.
